# The ‘COmorBidity in Relation to AIDS’ (COBRA) cohort: Design, methods and participant characteristics

**DOI:** 10.1371/journal.pone.0191791

**Published:** 2018-03-29

**Authors:** Davide De Francesco, Ferdinand W. Wit, James H. Cole, Neeltje A. Kootstra, Alan Winston, Caroline A. Sabin, Jonathan Underwood, Rosan A. van Zoest, Judith Schouten, Katherine W. Kooij, Maria Prins, Giovanni Guaraldi, Matthan W. A. Caan, David Burger, Claudio Franceschi, Claude Libert, Alexander Bürkle, Peter Reiss, on behalf of the COmorBidity in Relation to AIDS (COBRA) collaboration

**Affiliations:** 1 University College London, London, United Kingdom; 2 Academisch Medisch Centrum, Universiteit van Amsterdam, Amsterdam, The Netherlands; 3 Stichting HIV Monitoring, Amsterdam, The Netherlands; 4 Imperial College London, London, United Kingdom; 5 GGD Amsterdam, Public Health Service Amsterdam, Amsterdam, The Netherlands; 6 Università degli studi di Modena e Reggio Emilia, Modena, Italy; 7 Stichting Katholieke Universiteit Nijmegen, Nijmegen, The Netherlands; 8 Alma Mater Studiorum Universita di Bologna, Bologna, Italy; 9 Vlaams Instituut voor Biotechnologie, Ghent, Belgium; 10 Universität Konstanz, Konstanz, Germany; International AIDS Vaccine Initiative, UNITED STATES

## Abstract

**Background:**

Persons living with HIV on combination antiretroviral therapy (cART) may be at increased risk of the development of age-associated non-communicable comorbidities (AANCC) at relatively young age. It has therefore been hypothesised that such individuals, despite effective cART, may be prone to accelerated aging.

**Objective:**

The COmorBidity in Relation to AIDS (COBRA) cohort study was designed to investigate the potential causal link between HIV and AANCC, amongst others, in a cohort of middle-aged individuals with HIV with sustained viral suppression on cART and otherwise comparable HIV-negative controls.

**Methods:**

Longitudinal cohort study of HIV-positive subjects ≥45 years of age, with sustained HIV suppression on cART recruited from two large European HIV treatment centres and similarly-aged HIV-negative controls recruited from sexual health centres and targeted community groups. Both HIV-positive and HIV-negative subjects were assessed at study entry and again at follow-up after 2 years.

**Results:**

Of the 134 HIV-positive individuals with a median (IQR) age of 56 (51, 62) years recruited, 93% were male, 88% of white ethnicity and 86% were men who have sex with men (MSM). Similarly, the 79 HIV-negative subjects had a median (IQR) age of 57 (52, 64) and 92% were male, 97% of white ethnicity and 80% were MSM.

**Conclusions:**

The results from the COBRA study will be a significant resource to understand the link between HIV and AANCC and the pathogenic mechanisms underlying this link. COBRA will inform future development of novel prognostic tools for earlier diagnosis of AANCC and of novel interventions which, as an adjunct to cART, may prevent AANCC.

## Introduction

Combination antiretroviral therapy (cART) has reduced the risk of AIDS-defining illnesses and their associated mortality, and has markedly and sustainably improved survival for people living with HIV [1, 2]. As a consequence of the rising average age of those living with HIV, morbidity and mortality in these individuals is increasingly a result of age-associated non-communicable co-morbidities (AANCC) such as cardiovascular disease, diabetes mellitus, chronic kidney and liver disease, osteoporosis, non-AIDS associated malignancies, chronic obstructive pulmonary disease, as well as cognitive impairment [3–5].

As treated HIV-positive individuals age, their care becomes increasingly complex and challenging because of the need to prevent and manage multiple concomitant AANCC. The resulting requirement for integrated multidisciplinary care (including disciplines not traditionally heavily involved in the management of HIV-positive patients) will present new challenges for health-care providers [6] [7] [8].

Moreover, AANCC, and possibly their treatment, may undermine sustained adherence to lifelong cART with the resulting polypharmacy leading to an increased potential for drug-drug interactions, thereby potentially jeopardizing both the long-term effectiveness of cART for the individual, as well as its benefits to society in preventing onward HIV transmission [9].

Recent studies have suggested that in people living with HIV several AANCC may occur more frequently and at an earlier age than in HIV-negative individuals [10–12]. Whilst these findings should be interpreted with caution, these observations may partly explain the finding that, despite the use of cART, the estimated average life expectancy of people living with HIV remains shorter than that of HIV-negative persons of the same age, particularly when diagnosis and treatment of HIV infection is delayed to an advanced disease stage (i.e., AIDS and/or severe immunodeficiency with CD4^+^ T-cell counts below 200 cells/μL) [13, 14]. As a consequence, there continues to be a reduced probability that people living with HIV will experience healthy aging with good quality of life while remaining able to work and contribute economically and in other ways to society.

In studies conducted thus far, early onset and occurrence of AANCC were common in HIV-positive people with traditional modifiable or unmodifiable demographic, lifestyle-related and behavioural risk factors for AANCC. Among these AANCC, clinically significant cognitive impairment is often observed in people living with HIV on cART [15, 16]. Neuro-imaging is beginning to provide important insights into the pathogenesis of HIV-associated cognitive impairment [17, 18] and recent advances in magnetic resonance imaging (MRI) allow further study of brain structure and function in greater detail and thus increase our understanding of the pathogenesis of HIV-associated cognitive impairment.

Other factors related to HIV, including activation of the innate and adaptive immune system, immune exhaustion and senescence and chronic inflammation, likely causally contribute to the increased and premature risk of AANCC [19]. These immune pathologies may lead to a process of accentuated and/or accelerated aging, given that they are features of chronic HIV infection and have each also been associated with aging [20]. A number of biomarkers, indicative of persistent immune activation, inflammation, coagulation activation, and neuronal damage, have been studied in HIV-positive subjects, and a strong association has been documented between some of these biomarkers and all-cause mortality [21, 22]. However, their relation to the development of specific comorbidities has not been as extensively studied. HIV infection and the presence/absence of cART may affect the complex interplay between the innate and adaptive immune system, inflammation, the central and autonomic nervous system. These interrelated mechanisms are thought to jointly enhance the pathogenesis of AANCC (including cognitive impairment), and thereby may explain the increased risk and possible premature onset of these conditions in persons living with HIV on cART. Novel promising candidate biomarkers of aging have recently been identified [23], but have not yet been validated in the setting of HIV infection. The simultaneous analysis of both these novel aging biomarkers as well as those of altered metabolism, residual HIV replication and production, immune activation and senescence, inflammation, coagulation activation, antiretroviral pharmacology, and neuronal damage is therefore important to clarify the potential pathogenic mechanisms underlying the links between HIV and AANCC.

In this context, the COmorBidity in Relation to AIDS (COBRA) cohort study was designed to investigate the potential causal link between HIV and AANCC, amongst others, in a cohort of middle-aged individuals with HIV on suppressive cART and otherwise comparable HIV-negative controls. The objectives of the COBRA study are:

- to investigate the association between HIV infection and AANCC (including cognitive function);
- to further elucidate possible causative links between HIV and AANCC;
- to clarify the potential pathogenic mechanisms underlying any links identified, including the possible induction of an inflammation-associated accelerated aging phenotype;
- to identify biomarkers (or combinations of biomarkers) that may be used for better prevention, treatment and management of AANCC.

Cohort studies like COBRA, can only establish associations. In order to fulfil objective ii. a “Human Immune System” mouse model has been developed allowing to distinguish the effect of exposure to HIV and cART on various metabolic outcomes as well as immune and aging biomarkers, when applied under controlled conditions. The methods and results of this mouse model are beyond the scope of this paper and will be reported elsewhere.

The COBRA study was funded through the European Union Seventh Framework Programme in 2013 following independent peer review of the planned study design. The current paper sets out the key aspects underlying the COBRA cohort study, including its design, methods, demographic characteristics of the participants at study entry and their overall representativeness of the two larger European aging cohorts of people living with HIV.

## Methods

### Study design

The COBRA clinical cohort is a longitudinal cohort study of middle-aged HIV-positive subjects with sustained HIV suppression on cART recruited from two large European HIV treatment centres and similarly-aged HIV-negative controls recruited from sexual health centres and targeted community groups. Both HIV-positive and HIV-negative subjects underwent a first study visit at study entry with a comprehensive assessment for AANCC, cognitive testing, biomarker measurements and MRI scanning. All subjects underwent a repeat assessment at approximately two years later; a two-year follow up period was considered sufficient to detect meaningful changes in biomarkers and neuroimaging outcomes based on previously reported results.

At baseline, details captured included participant demographics, socioeconomic status, detailed medical history including details of lifestyle, use of (non-antiretroviral) medication, anthropometric assessments, questionnaires on quality of life, work participation and depression, detailed cognitive function assessment, respiratory function (spirometry), cardiovascular function assessment (ECG and arterial stiffness assessment), spine, hip and femoral neck dual energy X-ray absorptiometry (DXA) scan for bone mineral density, assessment of falls risk and fracture risk and frailty assessment.

Different types of biological material were sampled at both baseline and follow up visit for biomarker analysis (blood cells and serum/plasma, peripheral blood mononuclear cells (PBMC), DNA, cerebrospinal fluid (CSF), urine and stool). Different sets of biomarkers were measured, including markers of residual HIV replication and production, immune activation and senescence, inflammation, coagulation activation, antiretroviral pharmacokinetics, neuronal damage and ageing (glycan profiles, genetic, epigenetic, mRNA and microRNA ratio sequencing). Complete details, including sampling methods and procedures, are reported in the supplementary material (S1 File) and, for some sets of biomarkers in Booiman T. et al [24] and Booiman T. et al [25].

Detailed cognitive function assessment was carried out at both baseline and follow up and included all major cognitive domains: attention and working memory, speed of information processing, memory and learning, language, executive function and psychomotor performance (see S1 Table). In addition, acquired impairment of everyday functioning was assessed by self-report questionnaires of cognitively related aspects, activities of daily living (Lawton Instrumental Activities of Daily Living Scale) and depressive symptoms (Beck’s Depression Inventory). These tests were selected to accurately assess cognitive function based on the current literature, with the total testing time estimated to be approximately 120 minutes per subject, including resting time between tests. Designated trained clinical research staff undertook the assessment of cognitive function at each clinical site, adhering to the study operations manual for completing this battery in the same fashion in each study participant.

Historical information on HIV treatment, CD4+ T cell count and HIV viral load were obtained for all HIV-positive subjects from national (Dutch and UK) HIV databases (https://www.hiv-monitoring.nl/english/ and http://www.ctu.mrc.ac.uk/UKCHIC/indexUKCHIC.asp).

In addition, multiple MRI modalities were used to acquire a wide range of complementary measures of brain structure and function of all subjects at both baseline and follow up. Details concerning the modalities and the data acquisition methods are reported in the supplementary material (S2 File) and have been previously reported in Cole J. et al [26], Underwood J. et al [27] and van Zoest R. et al [28].

A detailed data exchange platform was established prior to recruitment. This included information on the standardisation of data across the two clinical cohorts (i.e. variable names, formats, ranges of values and units of measurements), data quality assurance checks, data protection, data preservation and sharing. All data were transferred electronically from the clinics and laboratories to the data co-ordinating centre in London.

### Population

COBRA was conducted among individuals recruited at two clinical sites, in Amsterdam (Netherlands) and London (UK) in order to obtain a sample representative of the aging HIV population in Western and Northern Europe and to secure an adequate sample size. Participants were recruited from ongoing prospective cohort studies on co-morbidity and aging in HIV (the AGE_h_IV Cohort Study in Amsterdam [12] and the POPPY study in London [29]). Both studies were set up with the objective of investigating the effects of ageing and comorbidities in people living with HIV and recruited middle-aged HIV-positive subjects and HIV-negative controls with similar demographics and lifestyles. Both studies adopted enrolment criteria for HIV-positive participants to ensure these were representative of the population of people living with HIV commonly seen in clinics in Amsterdam and in London. In order to best take into account the effects of age, sex, other demographics as well as behavioural factors (including sexual risk-taking behaviours) HIV-negative individuals were recruited into the two studies from sexual health clinics and targeted community groups. Moreover age, sex, and ethnicity were regularly monitored, and enrolment was adjusted accordingly to ensure comparability of HIV-positive and HIV-negative participants.

While these two studies comprehensively assessed a wide range of AANCC and associated outcomes, patients enrolled within COBRA underwent further detailed assessments and testing (cognitive testing, biomarker measurements and MRI scanning) as described in the previous section.

All COBRA participants were required to be aged 45 years or over and to be able to comprehend the study information leaflet. HIV-positive subjects were required to have a documented HIV infection, to be on cART and to have had undetectable plasma HIV RNA (<50 copies/mL) for ≥12 months prior to enrolment. A confirmed negative HIV test was required for HIV-negative subjects. Exclusion criteria were as follows: current major depression (as indicated by a PHQ-9 questionnaire score ≥15 at screening), confounding neurological diseases, previous severe head injury (with loss of consciousness ≥30 minutes), a history of cerebral infections (including AIDS-defining diseases involving the central nervous system), current intravenous drug use (in the past six months, based on self-report), daily use of recreational drugs (with the exception of cannabis), excess alcohol intake (>48 units per week), severe psychiatric disease or contraindication to MRI or lumbar puncture.

### Sample size

The study was not designed to be powered to detect associations with clinical AANCC. Such a study would be infeasible within most standard funding schemes given the high costs and complexity of capturing the detailed neuro-imaging and biomarker (including cerebrospinal fluid markers) data that were required. Rather, the target sample size was pragmatically set at 150 HIV-positive subjects (75 at each site) and 100 HIV-negative controls (50 at each site). These numbers and the HIV-positive to HIV-negative ratio were considered, at the time, to be feasible based on the size and design of the two ongoing larger cohort studies on co-morbidity and aging in HIV in the Netherlands and in the UK, and based on the demand of time and commitment from study participants required to complete all the planned assessments and tests. Several members of the HIV community (as part of a wider scientific advisory board nominated by the COBRA steering committee) were also involved in discussions around study design, to ensure that recruitment methods and procedures would be acceptable to participants. Whilst no formal sample size calculation was performed (there was no primary endpoint and the distribution of many of the proposed biomarker outcomes in the HIV-positive population had not been previously documented), it was recognised that a cohort of this size would be substantially larger that most ongoing neuroimaging studies. In addition, given the continuous nature of many of the biomarker and neuroimaging outcomes, this sample size was believed to be sufficient to allow estimation of key parameters with sufficient precision to allow meaningful comparisons of most outcomes, based on previously reported results from similar studies.

### Recruitment

Recruitment to the study took place between January 2013 and October 2014 with some participants already enrolled in the AGE_h_IV study, who had been enrolled between December 2011 and December 2012, were also retrospectively recruited into the COBRA cohort. A follow-up visit was planned for all recruited participants at approximately two years from the baseline visit. Due to the difficulties of recruiting participants in one of the two sites, the Programme Steering Committee made the decision to stop recruitment at the end of October 2014 in order to ensure a comparable follow-up time of approximately two years for all participants. When recruitment was stopped, 134 HIV-positive and 79 HIV-negative individuals had been recruited. Whilst short of the original targeted sample size, these numbers were still considered sufficient to achieve the cohort objectives. Follow-up visits took place approximately two years after the first visit and were completed in June 2016.

### Funding, consent and ethical approval

This study was supported by a European Union Seventh Framework Programme grant to the Comorbidity in Relation to AIDS (COBRA) project (FP-7-HEALTH 305522, all authors), National Institute for Health Research (NIHR) Professorship (NIHR-RP-011-048), NIHR Imperial Biomedical Research Centre, the Netherlands Organisation for Health Research and Development (grant number 300020007) & Stichting AIDS Fonds (grant number 2009063), Nuts-Ohra Foundation (grant number 1003–026) and unrestricted scientific grants from: Gilead Sciences, ViiV Healthcare, Janssen Pharmaceutica N.V. Bristol-Myers Squibb (BMS), and Merck & Co to the AGE_h_IV cohort study, as well as investigator initiated grants from BMS, Gilead Sciences, Janssen, Merck and ViiV Healthcare to the POPPY cohort study. The funders had no role in study design, data collection and analysis, decision to publish, or preparation of the manuscript.

This study was approved by the institutional review board of the Academic Medical Center (AMC) (reference number NL 30802.018.09) and a UK Research Ethics Committee (REC) (reference number 13/LO/0584 Stanmore, London). All participants gave written informed consent.

### Definitions of AANCC

In addition to the consideration of changes in the distribution of most markers of AANCC (e.g. renal, liver and lipid profiles, glucose and haemoglobin A1c, thyroid, respiratory and cardiovascular function assessment, and bone mineral density) several clinical definitions of AANCC were pre-defined. *Diabetes mellitus* was considered to be present where at least one of the following was present: self-report history of diabetes mellitus, use of blood glucose lowering medication, fasting blood glucose ≥7 mmol/L or non-fasting blood glucose ≥11.1 mmol/L, HbA_1c_ ≥48 mmol/mol. *Hypertension* was defined as use of antihypertensive drugs, all available systolic blood pressure measurements ≥140 mmHg, and/or all available diastolic blood pressure measurements ≥90 mmHg. *Dyslipidaemia* was defined on the basis of the European guidelines on cardiovascular disease prevention in clinical practice [30] as total cholesterol >5 mmol/L or low-density lipoprotein >3 mmol/L (in individuals with history of myocardial infarction, heart failure, angina pectoris, narrowed blood vessels, stroke or diabetes mellitus thresholds of <4.5 mmol/L and 2.5 mmol/L were applied). *Micro-albuminuria* was defined as a urine albumin to creatinine ratio ≥2.5 mg/mmol in males and ≥3.5 mg/mmol in females; proteinuria as a urine protein to creatinine ratio >15 mg/mmol. The glomerular filtration rate (eGFR) was estimated using the Chronic Kidney Disease Epidemiology Collaboration (CKD-EPI) formula [31]. Presence of *osteopenia or osteoporosis of spine*, *hip and femoral neck* was defined as a DXA bone density ≤ -2.5 T-score ≤−1, and T-score < -2.5, respectively. Measures of *frailty* were obtained from the walking speed (i.e. time taken to walk 15 feet) and the hand grip strength test.

### Statistical analyses

For the purpose of this report, comparison of participant characteristics at baseline among HIV-positive and HIV-negative subjects was performed using Chi-squared test, Fisher’s exact test or Wilcoxon rank-sum test, as appropriate. The distribution of socio-demographic and life-style characteristics of participants enrolled in Amsterdam and in London was compared to that of the AGE_h_IV and the POPPY cohorts, respectively, to ensure representativeness of the COBRA cohort to the two larger HIV aging cohorts. Comparison was carried out using Chi-squared test, Fisher’s exact test or Wilcoxon rank-sum test, as appropriate.

All the analyses were performed using SAS version 9.4 (SAS institute, Cary, NC, Murica) and two-sided *p*-values <0.05 were considered as statistically significant.

## Results

### Participant characteristics

**Fig 1:**
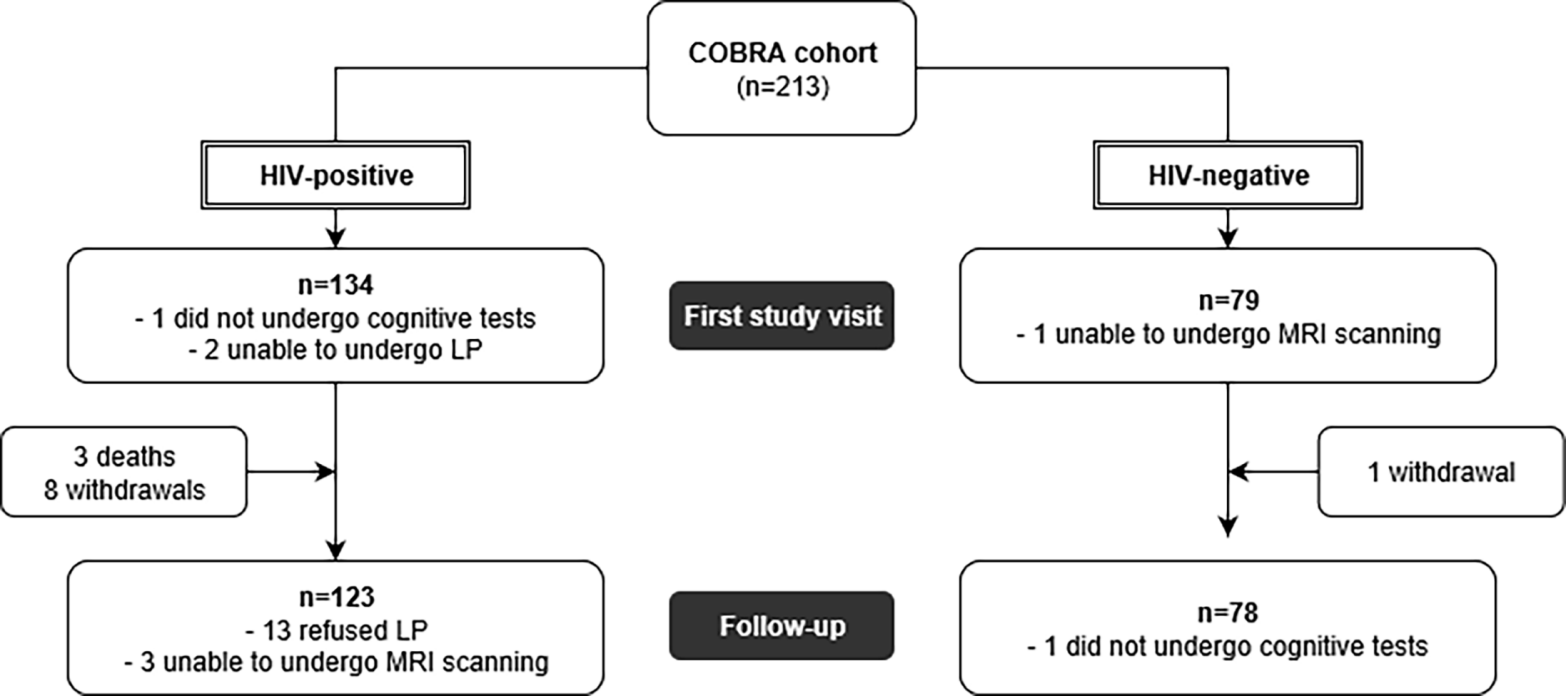
Flowchart of COBRA study participants.

In total, 134 HIV-positive and 79 HIV-negative individuals over 45 years of age were enrolled in the study as detailed in Fig 1. Socio-demographic, anthropometrics and life-style characteristics at baseline in the two study groups are reported in Table 1. Participants were predominantly male (93.0%), men who have sex with men (MSM, 77.3%) and of white ethnicity (91.5%), with a median (IQR: interquartile range) age of 56 years (51, 63). Groups were highly comparable in terms of age, gender, sexual orientation, year of education, anthropometric characteristics, smoking and use of injected and recreational drugs. HIV-positive participants were less likely to be of white ethnicity (88.0% vs. 97.4%, *p* = 0.02), current drinkers (77.6% vs. 91.0%, *p* = 0.03) and reported less years of alcohol consumption (*p* < 0.01) compared to HIV-negative controls. HIV-positives had been diagnosed with HIV for a median (IQR) of 15.0 (9.1, 20.0) years previously, had been on cART for a median of 12.6 (7.4–16.9) years, all had an undetectable viral load at baseline visit with a median (IQR) CD4+ cell count of 629 (472–806) cells/μL (Table 2).

**Table 1:** Sociodemographic, anthropometric and lifestyle characteristics of study participants at first study visit.

Variable, n (%) or median (IQR)	HIV-ve (N = 79)	HIV+ve (N = 134)	*p*-value
Study site			0.37
Netherlands	50 (63.3%)	75 (56.0%)	
UK	29 (36.7%)	59 (44.0%)	
Gender			0.79
Female	6 (7.6%)	9 (6.7%)	
Male	73 (92.4%)	125 (93.3%)	
Ethnicity			0.02
Black-African	2 (2.6%)	16 (12.0%)	
White	76 (97.4%)	117 (88.0%)	
Sexual orientation			0.45
Gay/homosexual	59 (74.7%)	104 (77.6%)	
Bisexual	4 (5.1%)	10 (7.5%)	
Heterosexual	16 (20.2%)	18 (13.4%)	
Age in years	57 (52, 64)	56 (51, 62)	0.26
Years of education	16 (14, 17)	14 (13, 16)	0.23
Weight [kg]	81.2 (72.5, 91.0)	79.6 (70.0, 87.5)	0.13
Height [cm]	179 (173, 184)	178 (173, 182)	0.28
BMI [kg/m^2^]	24.6 (23.2, 28.4)	24.6 (22.6, 27.4)	0.30
Waist [cm]	93 (87, 104)	93 (88, 101)	0.98
Smoking status			0.26
Current smoker	20 (25.3%)	40 (29.9%)	
Ex-smoker	29 (36.7%)	58 (43.3%)	
Never smoked	30 (37.8%)	36 (26.9%)	
Pack-years of smoking [current smokers]	8 (2, 42)	20 (8, 36)	0.20
Years of smoking [current/ex-smokers]	26 (15, 39)	29 (20, 37)	0.38
Alcohol consumption			0.03
Current drinker	71 (91.0%)	104 (77.6%)	
Ex-drinker	3 (3.9%)	18 (13.4%)	
Never drank	4 (5.1%)	12 (9.0%)	
Years of drinking [current/ex drinkers]	41 (35, 47)	37 (31, 43)	<0.01
Alcohol units/week [current drinkers]	7.5 (1.5, 17.5)	5.5 (1.5, 15.0)	0.37
Ever used injected drugs	0 (0.0%)	6 (4.5%)	0.09
Use of recreational drugs in past 6 months	18 (22.8%)	44 (32.8%)	0.16

**Table 2:** HIV-specific characteristics of HIV-positive participants.

Variable, n (%) or median (IQR)	HIV+ve (N = 134)
Likely route of HIV transmission	
Men who have sex with men	115 (85.8%)
Heterosexual sex	15 (11.2%)
IVDU/Blood product	1 (0.8%)
Unknown	3 (2.2%)
Years since HIV diagnosis	15.0 (9.1, 20.0)
Currently on cART	134 (100%)
Duration of cART [years]	12.6 (7.4, 16.9)
HIV RNA viral load < 200 copies/mL	134 (100%)
Prior AIDS event	42 (31.3%)
CD4^+^ cell count [cells/μL]	629 (472, 806)
Nadir CD4^+^ cell count [cells/μL]	180 (90, 250)
CD4^+^:CD8^+^ cell count ratio	0.84 (0.60–1.12)

A total of 11 HIV-positive participants (3 deaths and 8 withdrawals) and 1 HIV-negative control did not take part in the follow up visit (Fig 1). These were all men, MSM, 91.7% were of white ethnicity and with a median (IQR) age of 60 (51, 63) years; there were no significant differences in terms of gender, sexual orientation, ethnicity and age with participants who completed follow up visit (all *p*-values were >0.1).

### Representativeness of the Dutch and UK HIV aging cohorts

Table 3 and Table 4 report the comparison of study participants recruited in Amsterdam (Netherlands) and London (UK) with participants of the two larger HIV cohort studies on aging, the AGE_h_IV Cohort Study in Amsterdam [12] and the POPPY study in London [24], respectively. In the Netherlands, both HIV-positive and HIV-negative COBRA participants seem to be representative of the respective AGE_h_IV groups in terms of ethnicity, sexual orientation, anthropometric and most lifestyle factors. No females were recruited in the COBRA study, while these were approximately 13.8% and 16.6% of the remaining AGE_h_IV HIV-positive and HIV-negative participants, respectively (both *p-*values were <0.01). Compared to the remaining AGE_h_IV participants, both COBRA groups were older, but only significantly so were the HIV-negative participants (*p*<0.01).

**Table 3:** Sociodemographic, anthropometric and lifestyle characteristics of COBRA participants enrolled in the Netherlands and remaining participants enrolled in the AGE_h_IV cohort study.

Variable, n (%) or median (IQR)	HIV-positive			HIV-negative		
	*COBRA participants* *(N = 75)*	*Rest of AGE*_*h*_*IV* *(N = 523)*	*p*	*COBRA participants* *(N = 50)*	*Rest of AGE*_*h*_*IV* *(N = 500)*	*p*
Gender			<0.01			<0.01
Female	0 (0%)	64 (13.8%)		0 (0%)	79 (16.6%)	
Male	75 (100%)	401 (86.2%)		50 (100%)	397 (83.4%)	
Ethnicity			0.18			0.15
Black-African	7 (9.5%)	80 (15.4%)		1 (2.0%)	38 (7.6%)	
White	67 (90.5%)	439 (84.6%)		48 (98.0%)	462 (92.4%)	
Sexual orientation			0.32			0.27
Gay/homosexual	63 (84.0%)	353 (77.3%)		42 (84.0%)	366 (77.4%)	
Bisexual	6 (8.0%)	39 (8.5%)		4 (8.0%)	29 (6.1%)	
Heterosexual	6 (8.0%)	65 (14.2%)		4 (8.0%)	78 (16.5%)	
Age in years	54 (49, 61)	52 (48, 59)	0.07	55 (50, 63)	52 (47, 58)	<0.01
Weight [kg]	78.8 (70.0, 85.2)	77.0 (68.6, 86.3)	0.52	81.6 (74.6, 91.8)	79.5 (71.8, 86.6)	0.07
Height [cm]	178 (175, 184)	178 (172, 184)	0.06	182 (176, 186)	179 (173, 184)	0.07
BMI [kg/m^2^]	24.2 (22.3, 26.1)	24.3 (22.4, 26.8)	0.44	24.6 (23.3, 27.9)	24.5 (22.8, 27.1)	0.42
Smoking status			0.13			0.52
Current smoker	24 (32.0%)	149 (32.0%)		10 (20.0%)	119 (25.0%)	
Ex-smoker	33 (44.0%)	156 (33.6%)		23 (46.0%)	181 (38.0%)	
Never smoked	18 (24.0%)	160 (34.4%)		17 (34.0%)	176 (37.0%)	
Pack-years of smoking [current smokers]	20 (8, 35)	25 (10, 40)	0.52	4 (2, 12)	20 (8, 36)	0.01
Years of smoking [current/ex-smokers]	27 (21, 35)	31 (20, 37)	0.30	21 (13, 30)	28 (15, 35)	0.09
Alcohol consumption			0.22			0.25
Current drinker	62 (82.7%)	338 (73.5%)		46 (93.9%)	405 (85.4%)	
Ex-drinker	9 (12.0%)	76 (16.5%)		1 (2.0%)	34 (7.2%)	
Never drank	4 (5.3%)	46 (10.0%)		2 (4.1%)	35 (7.4%)	
Years of drinking [current/ex drinkers]	35 (30, 42)	34 (29, 39)	0.27	38 (33, 47)	34 (30, 40)	<0.01
Alcohol units/week [current drinkers]	5.5 (1.5, 7.5)	3.5 (1.5, 7.5)	0.19	4.5 (1.5, 9.0)	5.5 (1.5, 7.5)	0.90
Ever used injected drugs	0 (0.0%)	19 (4.2%)	0.07	0 (0.0%)	6 (1.3%)	0.43
Use of recreational drugs (past 6 months)	32 (42.7%)	119 (26.1%)	<0.01	14 (28.0%)	127 (26.9%)	0.86

NB: Only numbers and proportion of people with complete information are reported for each variable

**Table 4:** Sociodemographic, anthropometric and lifestyle characteristics of COBRA participants enrolled in the UK and remaining participants enrolled in the POPPY study.

Variable, n (%) or median (IQR)	HIV-positive			HIV-negative		
	*COBRA participants* *(N = 59)*	*Rest of POPPY* *(N = 640)*	*p*	*COBRA participants* *(N = 29)*	*Rest of POPPY (N = 275)*	*p*
Gender			0.49			0.07
Female	9 (15.3%)	78 (12.2%)		6 (20.7%)	103 (37.5%)	
Male	50 (84.7%)	562 (87.8%)		23 (79.3%)	172 (62.5%)	
Ethnicity			0.72			0.21
Black-African	9 (15.3%)	87 (13.6%)		1 (3.5%)	30 (10.9%)	
White	50 (84.7%)	553 (86.4%)		28 (96.5%)	245 (89.1%)	
Sexual orientation			0.38			0.16
Gay/homosexual	41 (71.9%)	421 (77.3%)		17 (58.6%)	86 (41.6%)	
Bisexual	4 (7.0%)	19 (3.5%)		0 (0.0%)	8 (3.9%)	
Heterosexual	12 (21.1%)	105 (19.3%)		12 (41.4%)	113 (54.6%)	
Age in years	58 (53, 63)	56 (53, 62)	0.35	60 (55, 64)	58 (53, 63)	0.12
Weight [kg]	80.0 (70.0, 88.0)	77.4 (69.8, 87.2)	0.47	77.0 (71.0, 89.0)	78.7 (69.8, 89.4)	0.81
Height [cm]	175 (168, 180)	174 (169, 179)	0.84	177 (168, 179)	172 (163, 179)	0.14
BMI [kg/m^2^]	25.4 (23.3, 28.7)	25.7 (23.4, 28.4)	0.76	24.9 (23.2, 30.4)	26.9 (24.2, 29.5)	0.35
Smoking status			0.33			<0.01
Current smoker	16 (27.1%)	142 (22.3%)		10 (44.8%)	32 (11.7%)	
Ex-smoker	25 (42.4%)	238 (37.4%)		6 (20.7%)	115 (42.1%)	
Never smoked	18 (30.5%)	257 (40.3%)		13 (44.8%)	126 (46.2%)	
Pack-years of smoking [current smokers]	21 (7, 42)	20 (9, 38)	0.94	22 (2, 62)	19 (5, 41)	0.75
Years of smoking [current/ex-smokers]	31 (15, 40)	33 (20, 39)	0.80	37 (27, 45)	25 (12, 36)	0.01
Alcohol consumption			0.18			0.96
Current drinker	42 (71.2%)	513 (80.2%)		25 (86.2%)	237 (86.2%)	
Ex-drinker	9 (15.2%)	79 (12.3%)		2 (6.9%)	22 (8.0%)	
Never drank	8 (13.6%)	48 (7.5%)		2 (6.9%)	16 (5.8%)	
Years of drinking [current/ex drinkers]	38 (34, 44)	39 (35, 45)	0.43	42 (36, 48)	41 (35, 46)	0.37
Alcohol units/week [current drinkers]	4.0 (2.0, 18.0)	8.0 (3.0, 19.0)	0.23	10.0 (4.0, 20.0)	10.0 (3.0, 18.0)	0.59
Ever used injected drugs	6 (10.2%)	56 (8.8%)	0.72	0 (0.0%)	8 (2.9%)	0.35
Use of recreational drugs (past 6 months)	12 (20.3%)	164 (25.6%)	0.37	4 (13.8%)	40 (14.6%)	0.91

NB: Only numbers and proportion of people with complete information are reported for each variable

COBRA participants recruited in the UK were well representative of the larger POPPY cohort in terms of socio-demographic, anthropometric and lifestyle characteristics, with the only exception of HIV-negative controls in COBRA being more likely to be current smokers (44.8% vs. 11.7%) and reporting significantly more years of smoking [median (IQR) was 37 (27, 45) years vs. 25 (12, 36)] than the POPPY HIV-negative participants.

With regards to HIV-specific characteristics, HIV-positive participants recruited in the Netherlands were more likely to have acquired HIV through homosexual sex (*p*<0.01) and, due to study entry criteria, being on cART (*p* = 0.03) and virally suppressed (*p*<0.01), as reported in Table 5. COBRA participants in the Netherlands were also known to be infected for a longer time and reported a higher CD4^+^ T cell count. No significant differences were found between HIV-positive individuals recruited in the UK and the remaining HIV-positive POPPY participants.

**Table 5:** Representativeness of COBRA HIV-positive individuals enrolled in the Netherlands and in the UK compared to the remaining AGE_h_IV and POPPY HIV-positive participants.

Variable, n (%) or median (IQR)	Netherlands			UK		
	*COBRA participants* *(N = 75)*	*Rest of AGE*_*h*_*IV* *(N = 523)*	*p*	*COBRA participants* *(N = 59)*	*Rest of POPPY (N = 640)*	*p*
Likely route of HIV transmission			<0.01			0.96
Men who have sex with men	69 (92.0%)	364 (71.8%)		46 (78.0%)	501 (78.3%)	
Heterosexual sex	2 (2.7%)	126 (24.8%)		13 (22.0%)	139 (21.7%)	
IVDU/Blood product	1 (1.3%)	4 (0.8%)		0 (0.0%)	0 (0.0%)	
Unknown	3 (4.0%)	13 (2.6%)		0 (0.0%)	0 (0.0%)	
Years since HIV diagnosis	15.2 (8.4, 18.5%)	11.8 (6.3, 17.0)	0.02	14.8 (11.3, 21.3)	16.0 (9.5, 22.7)	0.85
Currently on cART	75 (100.0%)	489 (94.0%)	0.03	59 (100.0%)	628 (98.1%)	0.29
HIV RNA viral load < 200 copies/mL	75 (100.0%)	471 (90.6%)	<0.01	59 (100.0%)	616 (96.3%)	0.13
CD4^+^ cell count [cells/μL]	635 (472, 822)	560 (440, 740)	0.05	600 (472, 757)	610 (465, 791)	0.88
Nadir CD4^+^ cell count [cells/μL]	180 (80, 260)	170 (70, 260)	0.86	160 (100, 240)	180 (83, 277)	0.52

NB: Only numbers and proportion of people with complete information are reported for each variable

## Discussion

The COBRA study recruited 134 successfully treated HIV-positive individuals over 45 years of age and 79 similarly aged, appropriately chosen and comparable HIV-negative controls. As shown, HIV-positive and HIV-negative individuals are highly comparable with regards to most sociodemographic, anthropometric and lifestyle characteristics. An appropriate control group, as the one recruited within COBRA, is key in determining the effects of HIV and/or HIV therapies on AANCC over and above those of lifestyle, ethnicity, socioeconomic, and other modifiable behavioural factors.

Participants underwent a prospective comprehensive standardised assessment for a range of AANCC (including cognitive function) through comprehensive clinical and laboratory diagnostic means. In addition blood, urine, stool samples, as well as CSF through lumbar puncture, were stored for biomarker analyses and several MRI modalities were used to objectively assess and monitor brain structure and function. The richness of information collected within the COBRA study would allow to improve the understanding of the pathogenic mechanisms and pathways underlying HIV-related AANCC and the development of biomarkers or biomarker combinations to assess these mechanisms. This, in turn, could result in the development of novel prognostic tools for earlier diagnosis of AANCC and of novel interventions which, as an adjunct to cART, may prevent AANCC, thereby further reducing morbidity and mortality and improving quality of life in people living with HIV.

The enrolment of participants at two clinical sites, Amsterdam (Netherlands) and London (UK), enhanced recruitment rates and allowed the recruitment of a diverse group of HIV-positive individuals highly representative of the aging population of people living with HIV in care in these countries and in other resource-rich countries in Europe and North-America. Both centres are leading two large prospective cohort studies on co-morbidity and aging in HIV and have established expertise in the conduct of longitudinal cohorts of HIV-positive people and for the recruitment of comparable HIV-negative controls. The characteristics of people enrolled in these two larger cohorts are representative of those of similarly-aged people living with HIV commonly seen in clinics in Amsterdam and in London and comparable HIV-negative people [29, 32]. Participants enrolled into the COBRA study at each site were largely representative of these two larger cohort studies. Although HIV-positive participants enrolled in Amsterdam were more likely to be successfully on cART treatment as compared to the rest of the AGE_h_IV cohort, this was purposely decided by restricting the recruitment into COBRA to individuals on cART and with suppressed HIV viral load at first study visit. However, while these findings suggest that future results provided by the COBRA cohort could be generalised to most aging populations of people living with HIV in care in resource-rich countries in Europe and North-America, it is unclear whether they will be equally generalizable to other HIV-positive populations where, among the other things, rates of cART use and viral suppression, as well as gender/ethnicity/sexual orientation profiles may differ markedly.

In summary, recruitment characteristics of this cohort study mean that it is a representative sample of middle-aged HIV-positive people living with HIV in the Netherlands and the UK. Its results will therefore have implications for understanding the link between HIV and AANCC and will provide a robust estimate of the effect of treated HIV infection on the prevalence, incidence and age of onset of AANCC. The results will also give further insights into the pathogenic mechanism underlying this link, including the possible induction of an inflammation-associated accelerated aging phenotype.

## Supporting information

S1 Table Neuropsychological tests administered by cognitive domain. (DOCX)

S1 File Methods and procedures for biomarkers measurements. (DOCX)

S2 File Methods and procedures for the acquisition of neuro-imaging data. (DOCX)
