# Supplementary material for: The ‘COmorBidity in Relation to AIDS’ (COBRA) cohort: Design, methods and participant characteristics
Source: PLoS One. 2018 Mar 29;13(3):e0191791. doi: 10.1371/journal.pone.0191791 (PMC5875743; doi:10.1371/journal.pone.0191791)
Supplement: S1 Table — (DOCX) [file pone.0191791.s001.docx]

**S1 Table.** **Neuropsychological tests administered by cognitive domain.**

| **Cognitive domain** | **Neuropsychological test** | **Scoring system** |
| --- | --- | --- |
| **Attention** | PASAT 3 | Total correct summations |
|  | WAIS-III Letter-number sequencing | Total correct sequences |
| **Executive function** | Trail Making Test-B | Total time to complete |
|  | Wisconsin CST | Number of total errors |
|  | Wisconsin CST | Number of perseverative errors |
|  | Wisconsin CST | Number of perseverative responses |
| **Language** | Category Fluency | Total number of animals in 1 minute |
|  |  | Total number of occupations in 1 minute |
|  | Letter Fluency | Total number of words, 1 minute for each of 3 letters |
| **Memory** | Rey Auditory Verbal Learning | Total recalled words trials 1-5 |
|  | Rey Auditory Verbal Learning | Total words recalled |
|  | WMS-IV Visual Reproduction | Immediate recall |
|  | WMS-IV Visual Reproduction | Delayed recall |
| **Motor function** | Grooved pegboard | Dominant hand: Time to complete |
|  | Grooved pegboard | Non-dominant hand: Time to complete |
|  | Finger tapping | Dominant hand: Median number of taps |
|  | Finger tapping | Non-dominant hand: Median number of taps |
| **Processing speed** | Trail Making Test-A | Time to complete |
|  | WAIS-III Digit Symbol | Total correct symbols |
|  | WAIS-III Symbol Search | Total correct symbols |
|  | Stroop colour-word test | Number of items completed |

PASAT = Paced auditory serial-addition task; WAIS-III = Wechsler Adult Intelligence Scale - Third Edition; WMS-IV = Wechsler Memory Scale - Fourth Edition.
