# Supplementary material for: The ‘COmorBidity in Relation to AIDS’ (COBRA) cohort: Design, methods and participant characteristics
Source: PLoS One. 2018 Mar 29;13(3):e0191791. doi: 10.1371/journal.pone.0191791 (PMC5875743; doi:10.1371/journal.pone.0191791)
Supplement: S1 File — (DOCX) [file pone.0191791.s002.docx]

**Residual HIV replication/transcription**

Cell associated unspliced HIV-RNA and total HIV gag-DNA have been measured using a seminested real-time PCR as described previously [1].

**Immune activation and inflammation**

Cryopreserved PBMC were stained with directly conjugated monoclonal antibodies for 30min at 4°C in the dark, to determine expression levels of different surface molecules on T cells and monocytes. Fluorescence was measured with the FACS Canto II (BD Biosciences). The proportion of cells expressing each marker were determined using FlowJo 7.6 (TreeStar, Ashland, OR, USA).

T cell differentiation was defined as the proportion of naïve (CD45RA+CD27+CCR7+), central memory (CD45RA-CCR7+CD27+), transitional memory (CD45RA-CCR7-CD27+), effector memory (CD45RA-CCR7-CD27-), and terminally differentiated effector memory (CD45RA+CCR7-CD27-) within the total CD4 or CD8 T cell population. T cell activation was defined as the proportion of cells that were positive for both CD38 and HLA-DR within the total CD4 or CD8 T cell population. T cell exhaustion was defined as the proportion of PD1 positive cells within the total CD4 or CD8 T cell population. T cell senescence was defined as the proportion of CD57 positive cells within the total CD4 or CD8 T cells population, the proportion of cells negative for both CD27 and CD28 within the total CD4 or CD8 T cells population, or the proportion of CD57 positive within the CD28-CD4+ or CD28-CD8+ T cell populations. The following directly conjugated monoclonal antibodies were used for cell surface marker staining on T cells: CD3 V500, CD4 PE-Cy7, HLA-DR Fitc, CD38 PE, CD28 PerCP Cy5.5, CD45RA PE-Cy7, CD8 Pacific Blue, CD57 APC, CCR7 PE, CD27 PerCP Cy5.5 (BD Biosiences, San Jose, CA, USA), CD27 APCeFluor780, CD4 APC eFluor780, and PD-1 PE (eBioscience, San Diego, CA, USA).

Monocyte activation status was defined by the expression of CD14 and CD16 in classical (CD14+CD16-), intermediate (CD14+CD16+) and non-classical (CD14+CD16++) monocyte subsets. In addition, the mean fluorescent intensity (MFI) of monocyte activation markers (CD163, CD32, CD64, CD38 and HLA-DR), T cell costimulatory molecules (CD40 and CD86) and adhesion molecules (CD91, CD11c and CX3CR1) were determined on the classical, intermediate and non-classical monocyte subsets. The following directly conjugated monoclonal antibodies were used for cell surface marker staining on monocytes: CD14 PE-Cy7, CD16 eFluor 450, CD32 PerCP-eFluor 710, CD11c APC (eBioscience, San Diego, CA, USA), CD163 AlexaFluor 488, CD86 PerCP (R&D Systems, Minneapolis, MN, USA), CX3CR1 PerCP-Cy5.5 (Biolegend, San Diego, CA, USA), HLA-DR V500, CD3 V500, CD4 PE-Cy7, HLA-DR Fitc, CD38 PE (BD Biosiences, San Jose, CA, USA), CD38 PE, CD91 PE (BD), CD40 APC-H7, CD64 APC-H7, CD8 Pacific Blue (BD Pharmingen, San Diego, CA, USA).

**Soluble markers of in plasma and/or CSF**

D-dimer and hsCRP concentrations were determined in fresh plasma samples using immunoturbidimetry (Sysmex CA-7000, Siemens, Munich, Germany and Cobas c702, Roche Diagnostics, Risch-Rotkreuz, Switzerland). I-FABP, sCD14 and sCD163 concentrations were determined in plasma and/or cerebrospinal fluid samples stored at −80°C using enzyme-linked immunosorbent assay (ELISA) (I-FABP, CD14 and CD163 DuoSet ELISAs, R&D systems, Minneapolis, Minnesota). Soluble CD16 concentrations were determined in plasma samples stored at −80°C by sandwich ELISA using mouse IgG1 antihuman CD16 3G8 mAb (BD, Pharmingen) as capture antibody, FITC-conjugated mouse IgG antihuman CD16 DJ130c mAb (Dako) as detection antibody and sheep IgG (Fab fragment) anti-FITC conjugated with HRP (Roche) as enzyme-linked secondary antibody as described elsewhere (21541219). Neopterin concentrations were measured in plasma and CSF stored at −80°C by enzyme-linked immunosorbent assay (BRAHMS Diagnostics/Thermo Fisher, Henningsdorf, Berlin, Germany). Tryptophan and kynurenine concentrations were determined in plasma and CSF stored at −80°C by high-performance liquid chromatography. The tryptophan to kynurenine ratio was calculated as index of tryptophan breakdown. TNFα, IP-10/CXCL10, MIP1α/CCL3, IL-6, MCP1/CCL2, MIG/CXCL9 and RANTES/CCL5 concentrations were analysed in plasma and CSF stored at −80°C by human magnetic luminex screening assay (LXSAHM-1 and LXSAHM-6, R&D Systems, Minneapolis, MN, USA).

NFL concentrations in CSF samples stored at −80°C were measured by sandwich ELISA (NF-light ELISA kit; UmanDiagnostics AB, Umeå, Sweden). Aβ1-42, p-tau, and t-tau were measured using ELISA as previously described [2] [3] [4].

**Viral load in plasma and cerebrospinal fluid**

CSF and plasma HIV RNA copy number was measured in stored CSF (−80°C) and fresh plasma samples using the Abbott RealTime M2000 assay (Abbot, Chicago, USA) with a lower limit of detection of 40 copies/mL.

**CMV antibody titers**

CMV total antibody titers and high avidity antibody titers were measured by ELISA-VIDITEST anti-CMV-IgG and IgG avidity (VIDIA, Praha, Czech Republic) according to the manufacturer’s instruction. For quantification a standard curve was prepared by serial dilution of plasma from a known CMV seropositive individual.

**Glycan profiles**

The N-glycans present on the proteins in serum were released, labelled, and analysed as by DSA-FACE technology, as described previously [5]. Data were analysed with the GeneMapper v3.7 software (Applied Biosystems, Foster city, CA).

**Other biomarkers of aging**

The carotenoids lutein, zeaxanthin, β-cryptoxanthin, lycopene, and α-/β-carotene, α-/γ-tocopherol, and retinol in plasma were simultaneously determined by HPLC with UV and fluorescence detection as previously described [6].

Alpha-2-macroglobulin in plasma was measured on the autoanalyzer (LX20 or DxC 800, Beckman-Coulter, Woerden, The Netherlands) by an immunoturbimetric method using reagents from Dialab, Wiener Neudorf, Austria as described by Jansen et al. [7]. Dehydroepiandrosterone sulfate, ferritin (female only), and prostate specific antigen (male only) in plasma were analyzed using an immuno-analyzer (Access-2, Beckman–Coulter, Woerden, The Netherlands) [7].

ELOVL2 and FHL2 DNA methylation in purified PBMC were analyzed using the Agena Bioscience’s EpiTYPER® DNA methylation analysis technology [8].

**Pharmacology**

Plasma and CSF drug concentrations of all licensed antiretroviral agents were analysed using validated HPLC/UV and LC/MS/MS techniques which were either published methods [9] [10] [11] or modifications of existing methods. All plasma assays are externally validated through participation in a proficiency testing program [12] [13]. Assay performance characteristics can be found in the cited papers.

**References**

1. Pasternak AO, Lukashov VV, Berkhout B. Cell-associated HIV RNA: a dynamic biomarker of viral persistence. Retrovirology. 2013;10(1):41.

2. Vanderstichele H, Blennow K, D’Heuvaert N, Buyse M-A, Wallin A, Andreasen N, et al. Development of a Specific Diagnostic Test for Measurement of β-Amyloid (1-42)[βA4 (l-42)] in CSF. Progress in Alzheimer’s and Parkinson’s Diseases: Springer; 1998. p. 773-8.

3. Blennow K, Wallin A, Ågren H, Spenger C, Siegfried J, Vanmechelen E. Tau protein in cerebrospinal fluid. Molecular and Chemical Neuropathology. 1995;26(3):231-45.

4. Vanmechelen E, Vanderstichele H, Davidsson P, Van Kerschaver E, Van Der Perre B, Sjögren M, et al. Quantification of tau phosphorylated at threonine 181 in human cerebrospinal fluid: a sandwich ELISA with a synthetic phosphopeptide for standardization. Neuroscience letters. 2000;285(1):49-52.

5. Vanhooren V, Laroy W, Libert C, Chen C. N-Glycan profiling in the study of human aging. Biogerontology. 2008;9(5):351.

6. Weber D, Stuetz W, Bernhard W, Franz A, Raith M, Grune T, et al. Oxidative stress markers and micronutrients in maternal and cord blood in relation to neonatal outcome. European journal of clinical nutrition. 2014;68(2):215-22.

7. Jansen E, Beekhof P, Cremers J, Weinberger B, Fiegl S, Toussaint O, et al. Quality control data of physiological and immunological biomarkers measured in serum and plasma. Mechanisms of ageing and development. 2015;151:54-9.

8. Bacalini MG, Deelen J, Pirazzini C, De Cecco M, Giuliani C, Lanzarini C, et al. Systemic Age-Associated DNA Hypermethylation of ELOVL2 Gene: In Vivo and In Vitro Evidences of a Cell Replication Process. The Journals of Gerontology Series A: Biological Sciences and Medical Sciences. 2016:glw185.

9. Droste J, Aarnoutse R, Burger D. Determination of emtricitabine in human plasma using HPLC with fluorometric detection. Journal of liquid chromatography & Related Technologies. 2007;30(18):2769-78.

10. Verweij-van Wissen C, Aarnoutse R, Burger D. Simultaneous determination of the HIV nucleoside analogue reverse transcriptase inhibitors lamivudine, didanosine, stavudine, zidovudine and abacavir in human plasma by reversed phase high performance liquid chromatography. Journal of chromatography B. 2005;816(1):121-9.

11. Droste J, Verweij-van Wissen C, Burger D. Simultaneous determination of the HIV drugs indinavir, amprenavir, saquinavir, ritonavir, lopinavir, nelfinavir, the nelfinavir hydroxymetabolite M8, and nevirapine in human plasma by reversed-phase high-performance liquid chromatography. Therapeutic drug monitoring. 2003;25(3):393-9.

12. Burger D, Krens S, Robijns K, Aarnoutse R, Brüggemann R, Touw D. Poor performance of laboratories assaying newly developed antiretroviral agents: results for darunavir, etravirine, and raltegravir from the international quality control program for therapeutic drug monitoring of antiretroviral drugs in human plasma/serum. Therapeutic drug monitoring. 2014;36(6):824-7.

13. Burger D, Teulen M, Eerland J, Harteveld A, Aarnoutse R, Touw D. The international interlaboratory quality control program for measurement of antiretroviral drugs in plasma: a global proficiency testing program. Therapeutic drug monitoring. 2011;33(2):239-43.
