# Supplementary material for: The ‘COmorBidity in Relation to AIDS’ (COBRA) cohort: Design, methods and participant characteristics
Source: PLoS One. 2018 Mar 29;13(3):e0191791. doi: 10.1371/journal.pone.0191791 (PMC5875743; doi:10.1371/journal.pone.0191791)
Supplement: S2 File — (DOCX) [file pone.0191791.s003.docx]

Multiple MRI modalities were used to acquire a wide range of complementary measures of brain structure and function. These included high-resolution structural (T1-MRI) to measure brain volume and cortical thickness, diffusion-MRI to investigate white matter microstructure, FLAIR to evaluate white matter lesions, magnetic resonance spectroscopy (MRS) to assess neural metabolites, resting state fMRI to investigate the brain network functional connectivity and arterial spin labelling (ASL) to assess cerebral perfusion. Different MRI scanner systems were used at each study site. In London a Siemens 3T Verio with 32-channel head coil was employed for both visits. In Amsterdam, initially data were acquired on Philips 3T Intera with an 8-channel head coil. During the baseline data acquisition phase, this was upgraded to a Philips 3T Ingenia, with a 16-channel head coil. The Ingenia scanner was used exclusively for follow-up visit scanning in Amsterdam.

Specific details of the acquisition parameters are as follows. In London: T1-MRI was a magnetisation-prepared rapid gradient-echo (MPRAGE) sequence; TE = 2.98ms, TR = 2300ms, TI = 900ms, flip angle = 9°, field-of-view = 256mm, 160 contiguous slices of 1mm thickness, voxel size = 1mm^3^. Diffusion-weighted images (for diffusion-MRI) were acquired along 64 non-collinear directions with b = 1000 s/mm^2^ and four images with b = 0 s/mm^2^, TE = 103ms, TR = 9500ms, field-of-view = 256mm, 64 contiguous slices, voxel size = 2mm^3^. High-resolution 3D fluid-attenuated inversion recovery (FLAIR) sequence: TE = 394ms, TR = 5000ms, TI = 1800ms, field-of-view = 250mm, 160 contiguous slices of 1mm thickness, voxel size = 1mm^3^. Single voxel ^1^H MRS data were acquired in three regions at baseline: frontal white matter, anterior cingulate cortex (ACC) and putamen. At follow-up, only frontal white matter and ACC were acquired. The sequence used 15mm^3^ isotropic voxels, manually positioned by trained radiographers, and 92 averages were acquired at TE = 30ms and TR = 2000ms for each region. Blood-oxygen level dependent (BOLD) response sensitive echo-planar imaging (EPI) was used to acquire resting state fMRI data for 10:06 minutes: TE = 30ms, TR = 2000ms, flip angle = 80°, field-of-view = 192mm, 35 interleaved slices of 3mm thickness with no gap, voxel size = 3mm^3^. Participants were instructed to remain still and not think of anything specific, while trying to stay awake. For ASL labelled and unlabelled images were acquired using a pulsed-ASL (pASL) sequence: TE = 11ms, TR = 2500ms, TI = 700/1800ms, flip angle = 90°, field-of-view = 192mm, 14 interleaved axial slices of 6mm thickness and a 29.5mm gap with the labelling slice.

In Amsterdam: T1-MRI was a sagittal Turbo Field Echo (T1-TFE) sequence; TE = 3.1ms, TR = 6.6ms, flip angle = 9°, field-of-view = 270mm, 170 contiguous slices of 1.2mm thickness, in-plane resolution = 1.1 x 1.1mm. Diffusion-weighted images were acquired 64 non-collinear directions with *b* = 1000s/mm^2^ and four images with *b* = 0 s/mm^2^. TE = 92ms, TR = 7081-9665ms, 64 contiguous slices, field-of-view = 270mm, voxel size = 2mm^3^. High-resolution FLAIR sequence: TE = 356ms, TR = 4800ms, TI = 1650ms, field-of-view = 250mm, 321 contiguous slices of 0.5 mm thickness, in-plane resolution = 1.05mm^2^. Single voxel ^1^H MRS data were acquired using point resolved spectroscopy (PRESS) in two regions at baseline: frontal white matter and putamen. At follow-up, only frontal white matter data acquired. The sequence used 15mm^3^ isotropic voxels, manually positioned by trained radiographers, and 64 averages were acquired at TE = 30ms and TR = 2000ms for each region. Resting state fMRI data were acquired using EPI SENSE mode for 7:00 minutes, with participants eyes closed: TE = 29ms, TR = 2000ms, flip angle = 80°, field-of-view = 240mm, 34 ascending axial slices of 3mm thickness with no gap, voxel size = 3mm^3^. For ASL labelled and unlabelled images were acquired using a pseudo-continuous ASL (pCASL) sequence: TE = 11ms, TR = 2500ms, label delay = 1650ms, flip angle = 90°, field-of-view = 240mm, 17 interleaved axial slices of 7mm thickness and a 20mm gap with the labelling slice.
